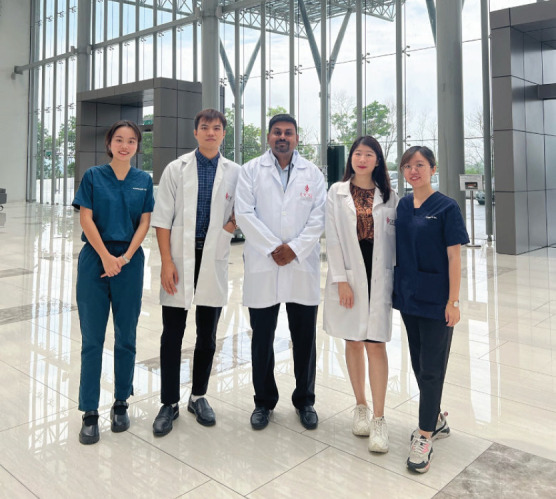# Changing tide of attire: Reflecting on the choice of clothing in primary care settings

**DOI:** 10.51866/mol.457

**Published:** 2023-10-27

**Authors:** Prasad Narayanan Haymond

**Affiliations:** 1 MD, FAFP, FRACGP, MMedEdu, Faculty of Medical & Health Sciences, USCI University, No. 2, Avenue, 3, Persiaran Springhill, Port Dickson, Negeri Sembilan, Malaysia. Email: haymondprasad@hotmail.com

Every year, as a primary care lecturer, I eagerly watch my first-year undergraduate students being adorned with white coats during the university’s White Coat Ceremony. This event marks the initiation of their journey towards becoming doctors and symbolises the beginning of their professional identity formation. However, the traditional white coat, which has long been a symbol of a doctor’s identity, is increasingly being replaced by business casual attire and surgical scrubs, especially in primary care clinics. This shift can be attributed in part to the rising generation of young doctors in Malaysia who view the traditional coat as passé. Some find the coat restrictive and uncomfortable owing to the country’s hot climate and have concerns about its weight and maintenance. Furthermore, the widespread adoption of the white coat by various professions has blurred its distinct association with the medical field, diminishing its unique identity for doctors. While I personally value the symbolism of the white coat and its role in fostering trust and professionalism, the declining tradition underscores a need to balance professional identity, patient trust and practical attire choices in contemporary medical practice.

**Figure:**